# The western Japan chaotic rainstorm disaster: a brief report from Hiroshima

**DOI:** 10.1186/s40560-018-0354-0

**Published:** 2018-12-17

**Authors:** Shinichiro Ohshimo, Takuma Sadamori, Nobuaki Shime

**Affiliations:** Department of Emergency and Critical Care Medicine, Graduate School of Biomedical and Health Sciences, Hiroshima University, 1-2-3 Kasumi, Minami-ku, Hiroshima, 734-8551 Japan

**Keywords:** Flooding, Landslide, Water supply, Unmanned aerial vehicle, Drone

## Abstract

The western Japan rainstorm disaster on July 6, 2018, was one of the most serious natural catastrophes in Japan, excluding earthquake events. Its main characteristics were severe and widespread flooding and landslides which cut off many areas, interrupting both traffic and telecommunication, and loss of clean water supply. We explored the utility of unmanned aerial vehicles to collect precise information on traffic disruption and damage to hospitals for patient rescue and for efficient allocation of resources. This visualized information was beneficial for determining rescue strategies. Lessons from this disaster and novel technologies could contribute to minimizing damage in future disasters.

## Background

A serious rainstorm event affected a broad area of western Japan during the night of July 6, 2018. More than 100 people died, 400 were injured, and 10,000 buildings were destroyed in this extreme weather event. The western Japan rainstorm disaster was one of the most serious natural catastrophes in the country, excluding earthquake events. The aim of our brief report was to provide the basic information of this rainstorm disaster having affected the western part of Japan.

## Clinical presentation

Hiroshima prefecture was the most seriously affected region; this may be associated with its geographical characteristics [1]. The region comprises mountainous areas with more than 5200 rivers flowing into low-lying districts with a thinly spread traffic network, as well as a city of 2.8 million people; the climate is moderate. The majority of Hiroshima prefecture is covered by decomposed granite soil, which consists of small grains and is less tolerable against heavy rain.

**Fig. 1:**
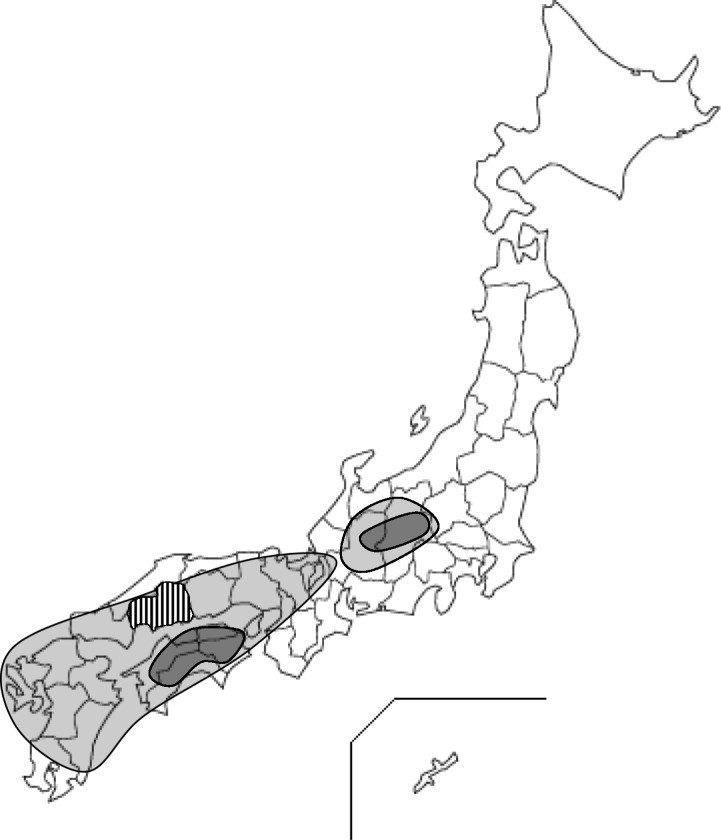
Map of Japan providing information of rainstorm area. Light gray indicates the area affected by the rain with a total amount of 400 to 800 mm. Dark gray indicates the area affected by the rain with a total amount of more than 800 mm. Striped area indicates Hiroshima Prefecture

**Fig. 2:**
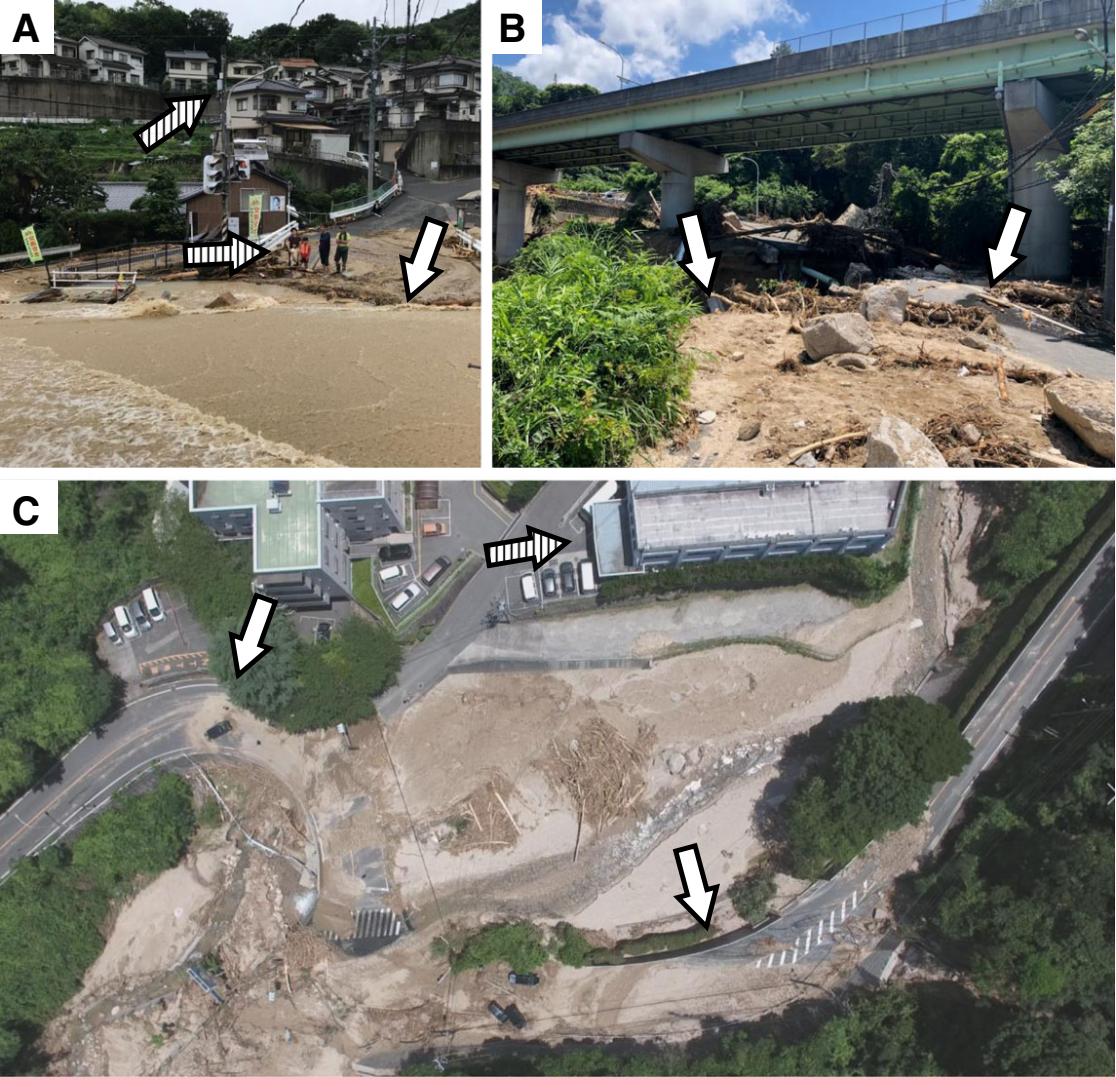
**a** View of an area isolated by floodwater on the second day of the disaster. Water flooded the roads (white arrow), and many elderly and young children living in houses built on the mountain slopes were stranded in the isolated area, unable to cross the flooded zone (striped arrows). **b** View under a land bridge of a road destroyed by landslides, taken by the unmanned aerial vehicle (PHANTOM4 Pro, DJI JAPAN Co., Ltd. Tokyo, Japan, flown by skilled drone operators). The clear visualization of the road damage (white arrows) was useful for planning the access to the isolated area across from the landslide. The high-definition video data were transmitted to the disaster management team by 4-GB live streaming using commercially available software. **c** Drone footage of an isolated hospital (striped arrow) and the road damaged by landslides (white arrows). This hospital was unable to provide information regarding the structural damage and loss of water, electricity, and gas supply

One of the major characteristics of this rainstorm disaster was the heterochronic onsets of multiple local disasters: severe and widespread flooding and landslides cut off many areas, interrupting both traffic and telecommunication, and loss of clean water supply. Heavy rain continued for as long as 3 days. The total amount of rain was 400 to 1000 mm, which was the largest amount during the last 50 years, as large as the total amount of rain during normal 2 months in this area (Fig. 1). A total of 1500 landslides heterochronously occurred. The severe flooding and landslides throughout the region caused many casualties and isolated many areas and hospitals (Fig. 2a).

Loss of water supply impaired hospital operation including hemodialysis and surgeries. We should have planned the managements including transportations of patients in the intensive care units. Disaster Medical Assistance Teams (DMATs) were established in Japan for rapid treatment during the acute phase of disasters [2]. However, the severe flooding and landslides throughout the region disrupted the DMATs’ road access to patients and hospitals.

Disruptions of cellular phone networks and internet connections also impaired the disaster management teams’ work [3]. Although precise information on the location and status of patients and hospital capacity in the affected area was essential for patient rescue and survival and for efficient allocation of resources, many hospitals did not have sufficient capacity or time to provide this information. To overcome this problem, we deployed an unmanned aerial vehicle (UAV), or drone, to collect information on traffic disruption and damage to hospitals [4] (Fig. 2b, c). For example, UAV images of an isolated hospital (Fig. 2c) and its surroundings revealed that the buildings, operations, and electricity of the hospital were not severely damaged and no patients were seriously injured; however, the water supply was terminated and access to the hospital was disrupted by rocks and landslides. This information was beneficial for determining rescue strategies such as prioritizing the removal of rocks and landslides with heavy machinery so that water supply could be restored, thus ensuring patients’ survival.

## Conclusions

We demonstrated the characteristics of the western Japan chaotic rainstorm disaster. Although the people and hospitals in western Japan are currently rebuilding after the devastating event, long-term continuous effort is necessary for full recovery. We believe that lessons from this disaster and novel technologies could contribute to minimizing damage in future disasters.

## Abbreviations

DMAT: Disaster Medical Assistance Team
UAV: Unmanned aerial vehicle
